# Clinical outcome of patients with epistaxis treated with nasal packing after hospital discharge

**DOI:** 10.1016/S1808-8694(15)30550-4

**Published:** 2015-10-19

**Authors:** Marina Faistauer, Ângela Faistauer, S. Grossi Rafaeli, Renato Roithmann

**Affiliations:** 1 MD. Graduated by the Medical School of the Lutheran University - Brazil, ULBRA; 2 5th Year Medical Student - PUCRS Medical School; 3 Otorhinolaryngologists - ULBRA; 4 MD; PhD, Adjunct Professor of Otorhinolaryngology - Medical School - ULBRA. Emergency Room Municipal Hospital Porto Alegre (HPS); ULBRA

**Keywords:** epistaxis, emergency medicine, recurrence

## Abstract

Epistaxis is a common clinical condition and in most public hospitals these patients received nasal packing and were admitted to the hospital as initial management strategies. However, little is known about the follow-up of these patients after they leave the hospital.

**Aim:**

To identify the clinical outcome of patients treated for epistaxis following discharge.

**Materials and Methods:**

We analyzed the results of questionnaires from patients hospitalized for non-traumatic epistaxis between March 2006 and March 2007.

**Study design:**

Cohort longitudinal.

**Results:**

Fifty-four of eighty-seven patients answered (62%). Epistaxis recurred in 37% of the patients. Of the patients who had recurrent bleeding, 70% were hypertensive, 35% were chronic users of acetylsalicylic acid, and 55% used tobacco. Forty per cent of the recurrences occurred in the first week after discharge, and fifty per cent needed to return to the emergency room. Seventy per cent of those who returned to the emergency room required a second treatment.

**Conclusions:**

Recurrence after epistaxis treatment is common and may occur soon after the initial discharge. Although our sample was small, this data suggests the need for a reevaluation of the current treatment mode of patients with epistaxis in the emergency rooms of public hospitals.

## INTRODUCTION

Epistaxis is defined as any bleeding arising from the nasal mucosa, it is the most common ENT emergency situation^1^, ^2^, ^3^, ^4^, ^5^, ^6^, ^7^, ^8^, ^9^, ^10^, ^11^, bearing a prevalence of about 10 to 12%^1^, ^4^, ^12^. Its Incidence is of 30 cases for every 100,000 inhabitants, and more than 87% of the patients seen by the ENT are admitted to a hospital^8^. This high frequency found is explained by the rich vascularization of the nose and paranasal sinuses, which receive blood supply from the internal and external carotid systems^12^.

Nasal bleeding can be caused by local or systemic factors. Among the systemic ones we can mention arterial high blood pressure, coagulopathy, blood disorders and the use of anticoagulant and anti-platelet adhesion factors. The most frequent local factors are: trauma (nasal fractures or finger manipulation), upper airway infections, cold and dry air breathing, nasal allergies, introduction of foreign bodies in the nasal cavity, septal perforation or deviation, tumors (juvenile nasoangiofibroma), blood vessel atherosclerosis at the Woodruff plexus and the Osler-Rendu-Weber disease or systemic telangiectasia^2^, ^4^, 13, 14, 15.

There are numerous treatment modalities, which go from simple manual compression, cauterization and packing, all the way to endoscopic or microscopic surgeries^2^.

The medical emergencies hospital where we carried out this study treats many patients with epistaxis of different causes and severity per day. Our routine in this public health service, which does not have an endoscopic assessment device, is to clean the clots and insert cotton balls with vasoconstriction agents followed by digital compression. Should the bleeding persist and the bleeding spot is not found for cauterization purposes, the nose is packed with foam and condom. Hospital admission is not a routine for this. Notwithstanding, elderly patients or those with uncompensated systemic diseases (e.g.: diabetes, hypertension, blood dyscrasia), or with recurrent epistaxis of difficult handling at home are treated in the hospital setting. Hospitalization time varies and, in some cases, hospital stay can be longer than 7 days.

After the bleeding stops, the packing is removed and the patient is observed for 12-24 hours. If the bleeding does not recur within this period, the patient is discharged and instructed to schedule an appointment with a physician or public health-care facility in order to be followed up. Therefore, we do not have enough information on the outcome in regards of the bleeding after hospital discharge. The need for further hospital visits and treatment is not known. In the literature we also do not have data regarding evolution after hospital discharge of those patients who were admitted to a hospital because of epistaxis.

This study aims at describing outcomes after hospital discharge of patients who had their noses packed and were hospitalized because of non-traumatic epistaxis in a public emergency room. More specifically, its aim is to check epistaxis recurrence rate, the time span between hospital discharge and recurrence and the need for further treatment.

## MATERIALS AND METHODS

The study was carried out in the department of otorhinolaryngology of a medical emergencies public hospital between March 2006 and March 2007. It is a historical cohort. All the medical charts from patients with non-traumatic epistaxis who received nasal packing and were admitted to this hospital were selected as part of the sample.

For data collection purposes, a detailed questionnaire was created, with special attention given to the type of treatment provided, hospital stay duration and recurrence after hospital discharge (Attachment 1). Through the hospital's data bank we were able to identify those patients admitted because of epistaxis during the period established. The charts were analyzed, looking for the identification data and the details regarding intra-hospital care. Afterwards, the patients and their respective guardians were contacted by phone call, and when authorized, after reading and signing the informed consent form, we employed the questionnaire. For those patients who did not have access to a telephone, we mailed an envelope with the questionnaire and a stamped envelope for them to mail the answers back to us.

The variables studied included: personal data, medical history, use of medication, hospital stays and post-hospitalization.

Data processing included the creation of a data bank and the analysis was carried out by the SPSS (Statistical Package for the Social Sciences) software, version 13.0 with the help of a statistician.

The quantitative variables were described by the average and standard deviation values (symmetrical distribution) or median and percentiles 25 – 75 (asymmetrical distribution). The categorical variables were described by means of the absolute and relative frequencies. In order to assess the categorical variables in relation to gender, recurrence and problem resolution with the new treatment. we employed the Pearson's chi-squared test or Fisher's Exact. In comparing the quantitative variables we used the t-Student test for independent samples (symmetrical distribution) or the Mann-Whitney test (asymmetrical distribution). The level of significance adopted was 5%, and values of p≤0.05 were considered statistically significant.

This study was approved by the Ethics in Research Committee of our institution, under protocol # 001.020617.07.5.

**Attachment 1 - Data collection spreadsheet**

**Patient's Chart Data:**

1. Chart number:
2. Name:
3. Age:
4. Gender:
5. Telephone/Address:
6. Profession:
7. Hospitalization duration:
8. Medical history: ( ) High blood pressure ( ) Diabetes ( ) Coronary disease ( ) Psychiatric disorder ( ) Asthma/CPOD ( ) Blood dyscrasia ( ) Other:
9. Use of medication: ( ) Acetyl salicylic acid ( ) Anticoagulant agent ( ) Anti-hypertensive ( ) NSAID ( ) other
10. Smoking: ( ) yes ( ) no
11. Drinking: ( ) yes ( ) no
12. Use of drugs: ( ) yes ( ) no
13. Habits: ( ) nasal pruritus ( ) cluster sneezes ( ) nasal obstruction ( ) chronic use of decongestant nasal drops ( ) nasal secretion
14. Past nasal surgeries: ( ) yes ( ) no

**Interview**

1. Was the nasal bleeding that caused hospitalization the first episode?
  ( ) Yes ( ) No
2. How many episodes had happened before? ( ) 1 ( ) 2 ( ) 3 ( ) 4 ( ) 5 or more
3. Was nasal packing done at the E.R.? ( ) Yes ( ) No
  Anterior or posterior? ____________________
4. Was nasal cauterization done at the E.R.? ( ) Yes ( ) No
  Chemical or electrical? ____________________
5. At the E.R was any other nose intervention done? ( ) Yes ( ) No
6. How long was the hospitalization? −− ____________________
7. After nasal packing removal and hospital discharge, was there another bleeding?
  ( ) Yes ( ) No
8. How many? ( ) 1 ( ) 2 ( ) 3 ( ) 4 ( ) 5 or more?
9. How long after hospital discharge without nasal packing you had another episode?
  ( ) 1 week or less ( ) 2 weeks ( ) 3 weeks ( ) 1 month
  ( ) 3 months ( ) 6 months ( ) 1 or + years
10. Did you have to return to the E.R? ( ) Yes ( ) No
11. How many times? ( ) 1 ( ) 2 ( ) 3 ( ) 4 ( ) 5 or more
12. Did you need another treatment? ( ) Yes ( ) No
13. Which? ( ) Cauterization ( ) Surgery ( ) Packing ( ) Embolization ( ) other
14. After this new treatment was the problem solved? ( ) Yes ( ) No

## RESULTS

There were losses because of difficulties to contact the cases. The initial sample was of 87 patients admitted because of non-traumatic epistaxis. We analyzed the data from questionnaires answered by 54 patients (62%). There was a 38% loss from the initial sample. Of all the losses, they happened because of: lack of knowing the telephone number and address (27%); because we only had the address (70%), did not answer the letters (45% from a total of 70%) or because the address was wrong (25% from a total of 70%); and one patient who was a street dweller (3%).

The mean age of the sample was 60.2 years (standard deviation of 15.4); with the extremes varying between 13 and 92 years, and it was more prevalent among males (53.7%). In relation to disorders related to a risk increase for epistaxis, 68.5% of the patients were hypertensive prior to hospital admission, 13% had diabetes, 14.8% had coronary disease, 9.3% had psychiatric disorder, 14.8% had COPD, 3.7% had blood dyscrasia and 25.9% had other diseases. Considering chronic use of medication, 31.5% used aspirin, 9.3% used oral anticoagulants, 14.8% used NSAIDs, 50% anti-hypertensive agents and 31.5% used other medication. Forty-eight percent of the participants were smokers and 9.3% drinkers of alcohol, and there was one case of drug addition among the patients.

Allergic rhinitis symptoms were prevalent among those interviewed; 40.7% of them reported nasal pruritus, 29.6% reported cluster sneezing; 24.1% nasal obstruction, 31.5% had nasal discharge and 14.8% chronically abused nasal drops. Thirteen percent of the sample had been submitted to nasal surgery before.

Sample characterization according to gender showed a higher mean age for men (62.5 years) when compared to women (57.5 years). Among men, 65.5% were retirees and the remaining 34.5% had jobs, while among women, 12% were retirees, 68% worked as volunteers and 20%, worked for a salary. Comparing gender and occupation regarding the latter data, we found statistically significant values, with p<0.001.

Systemic high blood pressure was the most prevalent disease associated with epistaxis for both genders, present in 72.4% of the men and 64% of the women. Regarding the most used medication, antihypertensive agents were also the most prevalent in both genders, being used by 51.7% of men and 48% of women. Most of the men (51.7%) and the women (44%) were smokers. Sixteen percent of the women and 10.3% of the men had been submitted to nasal surgery in the past. (Table 1)

**Table 1:** Sample characterization according to gender

Variables	Total (n=54)	Gender		P^d^
		Men (n=29)	Women (n=25)	
Age - Mean ± SD	60,2 ± 15,4	62,5 ± 13,2	57,5 ± 17,5	0,234^a^
Associated disorders - n(%)	…	…	…	…
HBP	37 (68,5)	21 (72,4)	16 (64,0)	0,711^b^
DM	7 (13)	6 (20,7)	1 (4,0)	0,108^c^
Coronary disease	8 (14,8)	5 (17,2)	3 (12)	0,711^c^
Psychiatric disorder	5 (9,3)	2 (6,9)	3 (12)	0,653^c^
Asthma/CPOD	8 (14,8)	2 (6,9)	6 (24)	0,125^c^
Blood dyscrasia	2 (3,7)	2 (6,9)	0 (0)	0,493^c^
Other disorders	14 (25,9)	6 (20,7)	8 (32)	0,526^b^
Medication - n(%)	…	…	…	…
Aspirin	17 (31,5)	10 (34,5)	7 (28,0)	0,828^b^
Anticoagulant	5 (9,3)	3 (10,3)	2 (8)	1,000^c^
Anti-hypertensive agent	27 (50)	15 (51,7)	12 (48)	1,000^b^
NSAID	8 (14,8)	4 (13,8)	4 (16)	1,000^c^
Other drugs	17 (31,5)	10 (34,5)	7 (28)	0,828^b^
Smoking - n(%)	26 (48,1)	15 (51,7)	11 (44)	0,769^b^
Alcohol abuse - n(%)	5 (9,3)	5 (17,2)	0 (0)	0,054^c^
Drug addiction - n(%)	0 (0)	0 (0)	0 (0)	…
Past nasal surgery - n(%)	7 (13)	3 (10,3)	4 (16)	0,692^c^

a t-student test for independent samples;

b Pearson's chi-squared test;

c Fisher's Exact Test

d p values mean the statistical difference between men and women for the variables analyzed.

For 50% of the patients, the hospital bleeding had been their first epistaxis episode. Regarding the others, 16.7% had had only one prior episode of nasal bleeding, while 13% had had 2 prior episodes, 5.6% 3 episodes and 14.8% – 5 or more anterior nasal bleedings prior to the hospital admission.

Regarding the nasal packing performed, most of the patients (98%) received anterior nasal packing. Anterior nasal packing had been done with a piece of foam inside a condom and lubricated with neomycin and bacitracin. Posterior nasal packing was carried out with a Foley Catheter.

Hospital stay duration was equivalent for both genders, and the epistaxis that caused this admission had been the first episode for 55.2% of the men and for 44% of the women. The patients with prior history of epistaxis, mostly males, had already suffered 5 or more episodes of epistaxis prior to the hospital admission (17.2%), while among females, most had had only one prior episode (20%) (Table 2). As far as treatment is concerned, all the men (considering 100 the male gender separately) and 96% of the women (considering 100% of female gender separately) were treated by anterior nasal packing.

**Table 2:** Dada regarding hospitalization according to gender

Variables	Total (n=54)	Gender		P
		Men (n=29)	Women (n=25)	
Hospitalization duration - Median (P25 - P75)	3 (2 – 4)	3 (2 – 4)	3 (2 – 4)	0,713^a^
Epistaxis during hospital stay was the 1st - n(%)	27 (50)	16 (55,2)	11 (44)	0,585^b^
# of episodes - n(%)c	…	…	…	…
1 prior episode of epistaxis	9 (16,7)	4 (13,8)	5 (20)	0,354^b^
2 prior episodes of epistaxis	7 (13)	4 (13,8)	3 (12)	…
3 prior episodes of epistaxis	3 (5,6)	0 (0,0)	3 (12)	…
4 prior episode of epistaxis	0 (0,0)	0 (0,0)	0 (0,0)	…
>=5 prior episodes of epistaxis	8 (14,8)	5 (17,2)	3 (12)	…

^c^ Only for those patients who answered no to the prior question.

**Graph 1:**
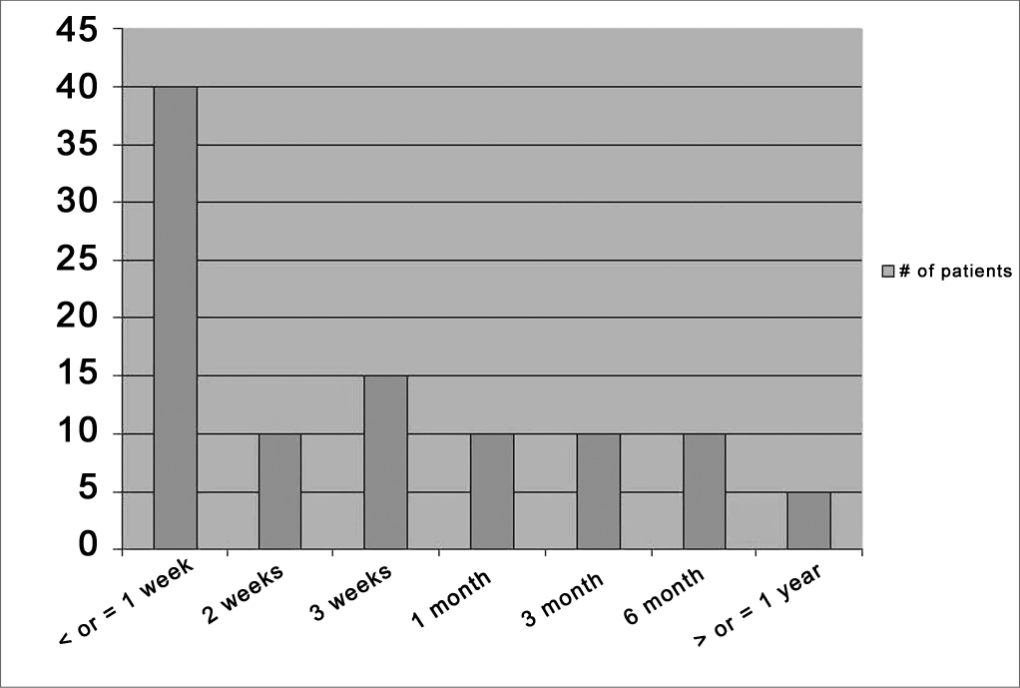
Time span between hospital discharge and the first episode of recurrence in the sample patients.

a Mann-Whitney test;

b Pearson's chi-squared test;

Epistaxis recurred in 37% of the patients after hospital discharge. Of these, all had had anterior nasal packing. Of all the women studied, 44% had recurrences, while among men this rate was 31%. Recurrence happened only once 75% of the cases, there were no important differences between the genders (Table 3). Of all the patients with epistaxis recurrence, 40% had a new bleeding episode in one week or less after hospital discharge (Graph 1).

**Table 3:** Post-hospitalization data according to gender

Variables	Total (n=54)	Gender		p
	Men(n=29)	Women (n=25)		
# of relapses (%)^a^	…	…	…	0,575^d^
1 recurrence	15 (75)	7 (77,8)	8 (72,7)	…
2 recurrence	1 (5)	0 (0,0)	1 (9,1)	…
3 recurrence	2 (10)	1 (11)	1 (9,1)	…
4 recurrence	1 (5)	1 (11)	0 (0,0)	…
>=5 recurrences	1(5)	0 (0,0)	1 (9,1)	…
# of returns to the E.R. - #(%)^b^	…	…	…	0,521^e^
Returned one timea	9 (81,8)	5 (100)	(66,7)	…
Returned 3 times	2 (18,2)	0 (0,0)	2 (33,3)	…
Required another treatment -#(%)	14 (25,9)	8 (27,6)	6 (24)	1,000^d^
Type of the new treatment #(%)^c^	…	…	…	0,971^d^
Cauterization	5 (35,7)	3 (37,5)	2 (33,3)	…
Nasal packing	7 (50)	4 (50,0)	3 (50,0)	…
Surgery	0 (0,0)	0 (0,0)	0 (0,0)	…
Embolization	0 (0,0)	0 (0,0)	0 (0,0)	…
Other	2 (14,3)	1 (12,5)	1 (16,7)	…
New treatment solved the problem #(%)^c^	10 (71,4)	6 (75)	4 (66,7)	1,000^e^

a Only for those patients who had recurrence of the epistaxis.

b Only for those patients who had to return to the E.R.

c Only for those patients who required retreatment.

d Pearson's chi-squared test;

e Fisher Exact Test

Fifty-five percent of the patients with epistaxis recurrence returned to the E.R because of bleeding, and 45% of these patients did not need to return to the hospital emergency ward (p<0.001). Of the patients who did return to the hospital emergency ward, 70% required a new treatment to control their nasal bleeding, and 30% did not (p<0.001). Of the treatment employed, because of recurrence, 50% underwent nasal packing, 35.7% were submitted to cauterization and 14.3% reported other modes of treatment. None of the patients were submitted to surgery or embolization. For 71.4% of the individuals the new treatment utilized was definitive (Table 3).

The mean age of the patients with epistaxis recurrence was 60.8 years; among those who did not have a recurrence, the mean age was 59.9 years. The hospital stay duration median value was equivalent for both groups – 3 days. Of the patients with recurrent epistaxis after hospital discharge, 70% were hypertensive and 20% had asthma/ COPD, while among those who did not recur, we found 67.6% with high blood pressure and 11.8% with asthma/CPOD. None of the patients with epistaxis recurrence had blood dyscrasia. As far as medication is concerned, all drugs analyzed had higher prevalence among those patients with a new episode of nasal bleeding: 35% used aspirin, 10% used anticoagulants, 55% anti-hypertensive agents, 15% NSAID and 30% used other medication. Fifty-five percent of the patients with recurrence were smokers, while 44.1% of those without recurrence were smokers. As to the symptoms of allergic rhinitis, there was no important difference regarding the occurrence of these relapses. Twenty percent of the patients with relapses had been previously submitted to nasal surgery, while 8.8% of those patients without recurrence had been submitted to surgery (Table 4). The bleeding which prompted the hospital admission had been the first for 55% of the patients who had relapses.

**Table 4:** Sample characterization according to the recurrence

Variables	Recurrence		p
	Yes (n=20)	No (n=34)	
Associated disorders - #(%)	…	…	…
HBP	14 (70,0)	23 (67,6)	1,000^a^
DM	1 (5,0)	6 (17,6)	0,239^b^
Coronary disease	2 (10,0)	6 (17,6)	0,695^b^
Psychiatric disease	3 (15,0)	2 (5,9)	0,347^b^
Asthma/COPD	4 (20,0)	4 (11,8)	0,450^b^
Blood Dyscrasia	0 (0,0)	2 (5,9)	0,525^b^
Other diseases	3 (15,0)	11 (32,4)	0,279^a^
Medication - n(%)	…	…	…
Aspirin	7 (35,0)	10 (29,4)	0,902^a^
Anticoagulant	2 (10,0)	3 (8,8)	1,000^b^
Anti-hypertensive	11 (55,0)	16 (47,1)	0,778^a^
NSAID	3 (15,0)	5 (14,7)	1,000^b^
Other drugs	6 (30,0)	11 (32,4)	1,000^a^
Smoking - n(%)	11 (55,0)	15 (44,1)	0,624^a^
Drinking - n(%)	1 (5,0)	4 (11,8)	0,640^b^
Drug use - n(%)	0 (0,0)	0 (0,0)	…
AR symptoms - n(%)	…	…	…
Nasal pruritus	8 (40,0)	14 (41,2)	1,000^a^
Cluster Sneezes	7 (35,0)	9 (26,5)	0,723^a^
Nasal obstruction	5 (25,0)	8 (23,5)	1,000^b^
Chronic use of DND	5 (25,0)	3 (8,8)	0,130^b^
Nasal discharge	6 (30,0)	11 (32,4)	1,000^a^
Past nasal surgery - n(%)	4 (20,0)	3 (8,8)	0,403^b^

a Person's chi-squared test;

b Fisher exact test

## DISCUSSION

The present study found a high rate of epistaxis recurrence after hospital discharge (37%). Moreover, it showed that most of the times the bleeding relapsed in the first week after hospital discharge and that about 20% of the patients returned to the hospital for another treatment. Considering only the patients with epistaxis recurrence, 55% returned to the emergency room because of bleeding and 70% of these required another treatment after this recurrence. We do not have literature data to compare with the results we found. Nevertheless, although it is a small sample - preventing us from doing further statistical analyses, 37% seem very significant. Explanations for this may stem from numerous factors, especially regarding the population making up this sample. Patients admitted for epistaxis usually have more severe and resistant signs and symptoms when compared to those who are not admitted. Moreover, most of them had systemic disorders and, since this is a public hospital, it does not have the proper conditions to treat and follow these patients up. Therefore, they do not use drugs to control high blood pressure, for example, and because of work demands, they can not rest enough during the nasal packing period.

Understand the outcomes after treatment provided in a health care institution is very important, because theoretically, it enables researchers to assess whether the treatment used is correct or if it can be improved. We observed that for most of the cases which relapsed, bleeding occurred in the first week after hospital discharge (40%), which challenges the current approach of discharging the patient 12 to 24 hours after packing removal in the institution analyzed. We speculate that if the patients were kept longer under observation and in bed rest, these recurrence rates could be lower, as well as the need for additional treatment. Nevertheless, such problem cannot be easily solved because of the lack of bed availability in public hospitals and the very need to save these beds for more critically ill patients. This is further reinforced by the fact that as they return to the emergency ward, a new packing solved the problem in 71% of the cases. Often times, when the patients returned with epistaxis after recent treatment by nasal packing, surgery was suggested. Nonetheless, in the hospital where this study was carried out, as it happens in most public hospitals, there is no proper material for embolization, nor surgery, and for this reason the patients are repacked.

Once the bleeding spot is identified by anterior rhinoscopy, vessel cauterization (chemical, electrical or laser cauterization) is the first treatment option for epistaxis. When such method is not sufficient to control bleeding, the patient may require anterior or posterior nasal packing^15^.

Nasal packing was introduced in the medical practice by Hippocrates and has been practiced until current days as a routine procedure in emergency wards^15^. Anterior nasal cavity packing is less efficient than cauterization, since it does not act directly on the bleeding vessel; however it applies uniform pressure on the entire mucosa^13^. The edema and inflammatory process resulting from the presence of a pack act preventing bleeding^15^. There are many nasal packs available, and the most commonly used are those by rayon or gauze. Other alternatives are the nasal packs created with glove fingers filled with gauze, regular household sponge inside a condom and Merocel packing^4^, ^14^. When bleeding is posterior or when anterior nasal packing is not enough, there is a need for posterior nasal packing. If the posterior packing is unable to control bleeding, or if upon its removal in a hospital setting after 48-72 hours there is bleeding recurrence, one must consider cauterization or endoscopic artery ligation.

Arterial embolization is more often used in nasal vascular tumors, such as the juvenile nasoangiofibroma, in the preoperative period, in order to reduce tumoral nasal flow during surgery. It can also be used in severe and persistent epistaxis which does not respond to clinical treatment^4^, 16, 17, 18.

Nasal packing with a foam piece inside a condom, or even a glove finger, is the most used method in most of the centers dealing with epistaxis^2^,^7^, 14, 15. It is a fast and easy treatment mode, and it is inexpensive - essential aspect of medical care in a public hospital setting. The duration of nasal packing is not absolutely defined in the literature; nonetheless, most ENTs leave it for at least 48 hours^2^,^4^, 14, 15. It does not seem to us that the premature nasal packing removal has been the cause for this high recurrence rate, but rather the factors discussed in the first paragraph (patients with more severe and clinically decompensated epistaxis). The treatment provided by the hospital of our study for inpatients with epistaxis is to keep them packed and in absolute bed rest until the bleeding subsides. Then, the packing is removed and the patient is discharged within 12 to 24 hours. This may be a factor to be considered. Another issue reported by this study is the need to invest in endoscopic surgery equipment. Since 20% of the cases return to the emergency room and need retreatment, including hospitalization. Surgery could, in the middle run, represent major savings for the public sector, knowing of its greater resolution power and lesser need for hospitalization time^1^, ^11^, 16, 17, 18, 19, 20.

Median hospitalization duration for the patients in our sample was 3 days, matching information found in the literature^3^, ^21^. The epistaxis episode during hospital stay was the first in 50% of the patients. We do not have data in the literature to compare ours with.

Epistaxis has a bimodal distribution in relation to age, with the first incidence peak during childhood and the next around 50 years and on^3^, 13, 14. We believe our study has shown only one peak involving elderly patients, because of the hospital stay factor which results from more severe epistaxis, usually due to systemic diseases - more prevalent in older individuals.

By our statistical analyses of the sample evaluated, we did not find differences between the patients with and without epistaxis recurrence; nonetheless, these results have to be interpreted very carefully, because of the small sample and consequently its small statistical power. However, some observations still apply. Sixty-nine percent of the patients in this sample were hypertensive. The literature shows a clear association between high blood pressure and epistaxis^5^; however, the cause and effect relationship is chalenged^22^. Other two risk factors for epistaxis which had high rates in this sample were smoking (48%) and the use of aspirin (31%). Literature shows that there is an association between the use of aspirin and epistaxis^4^, 13, 14, 15, and the risk of nasal bleeding increases in approximately two fold in those patients using such medication^22^. As far as smoking is concerned, it is mentioned as a risk factor for severe epistaxis^23^. The symptoms associated with allergic rhinitis presented high incidence in this sample, especially nasal pruritus (41%). Epistaxis can be triggered by itching, blowing the nose, coughing, sneezing, the use of decongestant nasal drops - factors are associated to nasal allergies^4^, 13, 14, 15.

Many can be the reasons for the high incidence seen with the technique used in our sample, one of them is that these more severely ill patients come to the emergency service and require hospitalization to treat not only the bleeding, but also comorbidities. Another aspect is that the condom with the foam piece inside (or sponge) usually fills better the lowermost portions of the nasal cavity. Some patients, in whom the bleeding comes from the upper nose may not have successful outcomes by this approach. And finally, we can speculate that the time required to remove the packing was too early.

One systematic mistake identified in this study happened in relation to the follow up time. The time between hospitalization and the patient's interview was not factored, in other words, interviewed patients in a longer period of time after the hospitalization epistaxis have a greater risk of recurrences in relation to our interviewees in less time. Another issue that must be highlighted associated with sample losses is the location of some cases and the response in others. These represented a major difficulty in this study, present at two moments: first, because of the lack of data that could allow patient access (telephone, address) in the medical charts; and at a second time, by the lack of individual interest in cooperating with the study, not answering the mail containing the questionnaire.

## CONCLUSION

The recurrence rate of non-traumatic epistaxis in patients who were packed and hospitalized in a public hospital of medical emergencies was 37%. The time span between discharge and recurrence in most of the cases happened in the first week after hospital discharge (40% of the cases) and about 20% of the cases returned to the emergency room for reassessment and treatment. Despite the sample being small, these data require a reassessment of the current model of epistaxis management in most public hospitals.
